# Simulation of Soil Temperature Dynamics with Models Using Different Concepts

**DOI:** 10.1100/2012/590287

**Published:** 2012-06-18

**Authors:** Renáta Sándor, Nándor Fodor

**Affiliations:** Centre for Agricultural Research, Hungarian Academy of Sciences, 2462 Martonvásár, Hungary

## Abstract

This paper presents two soil temperature models with empirical and mechanistic concepts. At the test site (calcaric arenosol), meteorological parameters as well as soil moisture content and temperature at 5 different depths were measured in an experiment with 8 parcels realizing the combinations of the fertilized, nonfertilized, irrigated, nonirrigated treatments in two replicates. Leaf area dynamics was also monitored. Soil temperature was calculated with the original and a modified version of CERES as well as with the HYDRUS-1D model. The simulated soil temperature values were compared to the observed ones. The vegetation reduced both the average soil temperature and its diurnal amplitude; therefore, considering the leaf area dynamics is important in modeling. The models underestimated the actual soil temperature and overestimated the temperature oscillation within the winter period. All models failed to account for the insulation effect of snow cover. The modified CERES provided explicitly more accurate soil temperature values than the original one. Though HYDRUS-1D provided more accurate soil temperature estimations, its superiority to CERES is not unequivocal as it requires more detailed inputs.

## 1. Introduction

Soil temperature (*T*_soil_) is one of the most important variables of the soil. It can significantly influence seed germination [1], plant growth [2], uptake of nutrients [3], soil respiration [4, 5], soil evaporation [6], and the intensity of physical [7], chemical [8, 9], and microbiological processes [10, 11] in the soil.

Solar radiation and air temperature are the main driving forces determining the soil temperature which is influenced by numerous other factors such as precipitation, soil texture, and moisture content as well as the type of surface cover (plant canopy, crop residue, snow, etc.) [12]. Yearly, monthly, or daily means of soil temperature measurements are frequently reported, but the variability of *T*_soil_ is similarly important [13]. In spite of this, at many meteorological stations only aboveground variables (e.g., air temperature) are observed, or the soil temperature sensors are installed at the station (close to the mast that supports other sensors and the data logger) and not at the plots of the experimental site which could make the measured data unrepresentative.

If soil temperature is not measured, several methods are available to calculate it using meteorological variables and other parameters. As the simple air-temperature-based methods (e.g., [14]) provided inadequate *T*_soil_ data, an improved formula was introduced that uses precipitation data [15] as well. There are three types of soil temperature models [16]: (1) empirical models that are based on statistical relationships between soil temperature at some depth and climatological and soil variables (e.g., [17]); (2) mechanistic models that focus on physical processes (radiative energy balance as well as sensible, latent, and ground-conductive heat fluxes) to predict the upper boundary temperature and estimate the temperature of deeper layers with Fourier's equation (e.g., [18]); (3) mixed empirical and mechanistic models that calculate the temperature of different soil layers based on physical principles of heat flow, but the boundary temperature at the soil surface must be provided empirically (e.g., [19]).

Since LAI (leaf area index) and soil water balance strongly influence the, soil temperature dynamics, soil temperature calculating methods function more precisely when those are integrated into hydrological [20, 21] or crop simulation models [22, 23]. The primary purpose of these models is to describe the processes of the very complex atmosphere-soil-plant system, including human activities, using mathematical tools and to simulate them with the help of computers.

The objectives of this paper are as follows: (1) presenting the effect of LAI on soil temperature; (2) comparison of an empirical and a mechanistic soil temperature model using measured data; (3) enhancing the performance of the empirical model.

## 2. Materials and Methods

Data of the agrometeorological station at Őrbottyán, Hungary were used in the study. The arenosol of the experiment site has the following characteristics: bulk density: 1.67 gcm^−1^; organic matter content: 0.91%; CaCO_3_ content: 5.1%; sand fraction: 86.3%; silt fraction: 8.3%; clay fraction: 5.4% [24]. Saturated hydraulic conductivity (Ks) and characteristic points of the soil water retention curve (SWRC) were measured with Guelph permeameter [25] and Eijkelkamp sand/kaolin box apparatus, respectively. pF-measurements were carried out with 100 cm^3^ undisturbed samples taken in 5 replicates. The van Genuchten parameters [26] of the SWRC were determined with Soilarium [27]. The above parameters characterize the 0–20 cm layer of the soil. In the 20–60 cm layer, the parameter values are practically the same except for the organic matter content which gradually decreases to zero with depth.

Soil temperature sensors (thermistor type, ±0.5°C accuracy, 0.1°C resolution) were installed at 5 different depths (5, 10, 20, 40, and 60 cm) at the centre of each 10 × 15 m test plot of an experiment with 8 parcels realizing the combinations of the fertilized, nonfertilized, irrigated, and nonirrigated treatments in two replicates. Temperature data were recorded every 15 minutes. A meteorological station was installed next to the experiment where precipitation, relative humidity, wind velocity and direction, global radiation, and air temperature were measured every 5 minutes in 2010 and 2011. In these two years, maize was grown at the site. LAI of every parcel in three-week intervals were determined by direct measurements. Three plants were cut out randomly at every observation time, and the area of the leaves was calculated with Montgomery's method [28].

Site-specific measured data were used as inputs for the CERES-Maize [29] crop simulation model as well as for the HYDRUS-1D [21] hydrological model. CERES is a daily-step deterministic model that simulates plant (assimilation, biomass accumulation, leaf area, dynamics and root growth) as well as soil (water, temperature and nutrient dynamics) processes using empirical equations. HYDRUS-1D is designed for simulating one-dimensional variably saturated water flow, heat movement, and the transport of solutes in the soil. It numerically solves the Richards equation for saturated-unsaturated water flow (including a sink term to account for water uptake by plant roots) and advection-dispersion type equations for heat and solute transport using Galerkin-type linear finite element schemes. Several studies proved the efficiency of both models [30, 31].

The soil temperature calculation module of CERES belongs to the empirical model group. When calculating the actual temperature (*T*_soil_^*i*^) at a given depth (*x*), this model takes into account that the upper soil layers absorb energy, and the heat needs time to reach the lower layers as in (1). The effect of the energy reaching the soil surface appears delayed and decreased in the lower soil layers. The extent of the delay and the decrease is a function of the actual average moisture content (Θ_avg_^*i*^) and the average bulk density of the topsoil (BD_avg_). The model assumes a sinusoidal annual course of the soil surface temperature that is modified by an additive term of a five-day moving average of a factor described by (2) as follows:

(1) $$T_{\text{soil}}^{i}\left(x\right)=\overset{Td}{\overset{︷}{T_{\text{avg}}+\left(\frac{T_{\text{amp}}\cdot \cos\left(0.0174\cdot (i-I)+x\cdot f_{1}\left(\Theta_{\text{avg}}^{i},{\text{BD}}_{\text{avg}}\right)\right)}{2}+DT^{i}\right)}}\cdot e^{x\cdot f_{2}(\Theta_{\text{avg}}^{i},{\text{BD}}_{\text{avg}})},$$

(2) $$\;DT^{i}=\frac{\sum_{j=i-4}^{i}\left(1-\text{ALB}\right)\cdot \left(T_{\text{mean}}^{j}+\left(T_{\max}^{j}-T_{\text{mean}}^{j}\right)\cdot \sqrt{0.03\cdot S_{\text{rad}}^{j}}\right)}{5}-T_{\text{avg}}-\frac{T_{\text{amp}}\cdot \cos\left(0.0174\cdot \left(i-I\right)\right)\;}{2},$$

where  *i* denotes the day of the year;  *I* equals 200 on the northern hemisphere, while it is 20 on the southern hemisphere. ALB is the albedo of the surface, *T*_avg_ and *T*_amp_ denote the average temperature and the average temperature difference of the site. *T*_mean_^*i*^, *T*_max⁡_^*i*^, and *S*_rad_^*i*^ denote the daily mean and maximum temperature as well as the daily global radiation on the *i*th day of the year, respectively. The term *Td* in (1) describes the delay of the effect of energy reaching the surface in deeper layers. The exponential term in (1) is more related to the heat capacity of the topsoil as it governs the decrease of the effect of the incoming energy at the surface in deeper layers.

The soil temperature calculation module of HYDRUS-1D belongs to the mechanistic model group. It numerically solves the convection-dispersion equation describing the one-dimensional heat transfer as follow: (3).

(3) $$\frac{\partial C_{s}\left(\Theta \right)T}{\partial t}=\frac{\partial }{\partial x}\left(\lambda \left(\Theta \right)\frac{\partial T}{\partial x}\right)-C_{w}\frac{\partial qT}{\partial x}-C_{w}\cdot S\cdot T.$$

*θ* is the volumetric water content; *λ* denotes the apparent thermal conductivity of the soil. *C*_*p*_and *C*_*w*_ are the volumetric heat capacities of the solid and the liquid phases, respectively. *S* is the sink term, and *q* is the Darcian fluid flux density. The apparent thermal conductivity can be expressed with (4) based on the work of de Marsily [32] as well as of Chung and Horton [33]:

(4) $$\lambda \left(\Theta \right)=b_{1}+b_{2}\cdot \Theta +b_{3}\cdot \Theta^{0.5}+\beta_{t}\cdot C_{w}\left|q\right|,$$

where *β*_*t*_ is the thermal dispersivity, while *b*_1_, *b*_2_, and *b*_3_ are empirical parameters that can be estimated by using the sand, silt, and clay content of the soil.

Initial conditions of the water flow domain were measured with TDR (IMKO TRIME-FM3) tube access probe in 10 cm increments in three replicates. The initial soil temperature was set to uniform 10°C in the whole profile. HYDRUS-1D requires the setting of boundaries conditions for solving the flow equations. For water flow, atmospheric boundary conditions with surface runoff and free drainage were prescribed at the upper and lower boundary, respectively. For heat flow, the temperature values at both boundaries were provided in the model input file.

**Figure 1:**
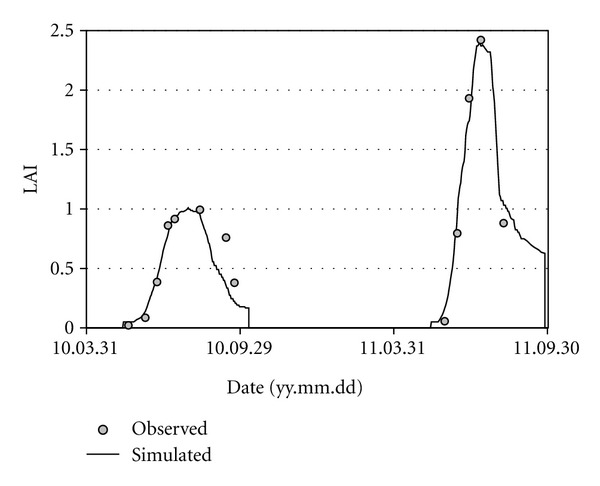
Observed and simulated leaf area index (LAI) values for the 1st parcel (fertilized, nonirrigated) of the experiment.

The parameters of CERES were calibrated by inverse modeling [34] so that the simulated LAI values would be in good agreement with the observed values (Figure 1). The obtained daily LAI values were used as inputs for HYDRUS-1D, as well. The measured and calculated soil temperature values were compared with simple graphical and statistical tools.

Originally, the user cannot alter the functions ((1) and (2)) of heat transport in CERES. Though it is an empirical model, it cannot be calibrated. In other words, it is postulated that it works for all soil types. A simple modification of one of its governing equations (1) is proposed to provide greater flexibility and the possibility of site-specific calibration for the following model: (5).

(5) $$T_{\text{soil}}^{i}\left(x\right)=Td\cdot e^{c\cdot x\cdot f_{2}(\Theta_{\text{avg}}^{i},{\text{BD}}_{\text{avg}})}.$$

By modifying the value of parameter *c* in (5), the amount of heat reaching the deeper soil layers could be adjusted. Though this parameter has no clear physical meaning, it most likely integrates the effect of soil organic matter, soil structure, and other implicit factors on soil-specific heat.

## 3. Results

**Figure 2:**
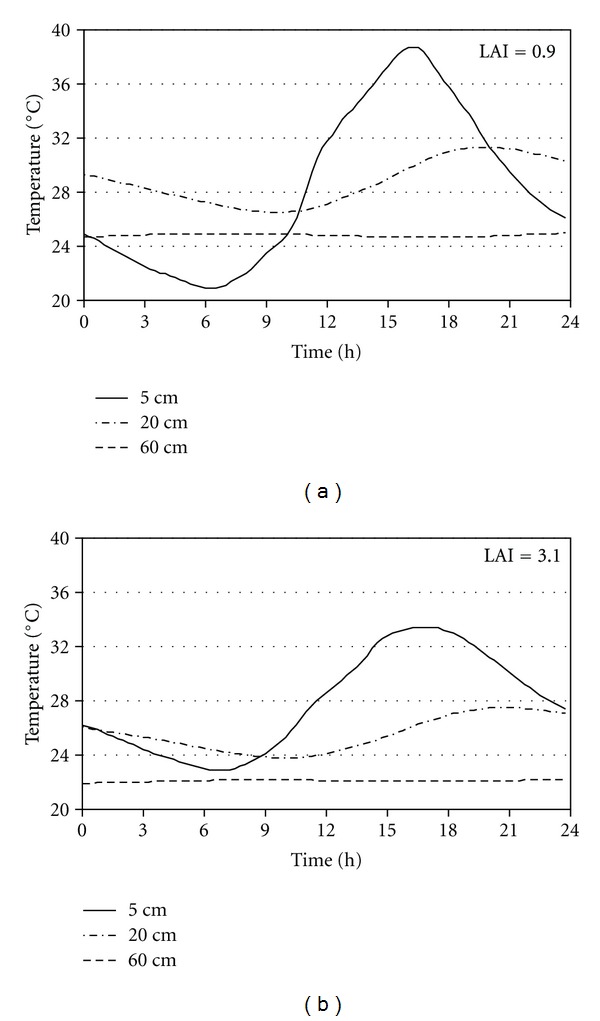
Effect of leaf are index (LAI) on the soil temperature dynamics at different depths in a non-fertilized (to the left) and in a fertilized parcel (to the right) on 14/07/2011 at Őrbottyán, Hungary.

Considerable differences in the leaf area indices were observed in different treatments of the experiment at the end of the canopy development in 2011 (Figure 2). Over 5°C, difference was observed in the daily maximum temperature at 5 cm depth, in the selected parcels. At 20 cm depth the observed difference was still explicitly greater (3.8°C) than the measurement error. The peek temperature at this depth occurred 6 hours later (at sunset) than the peek of the air temperature.

**Figure 3:**
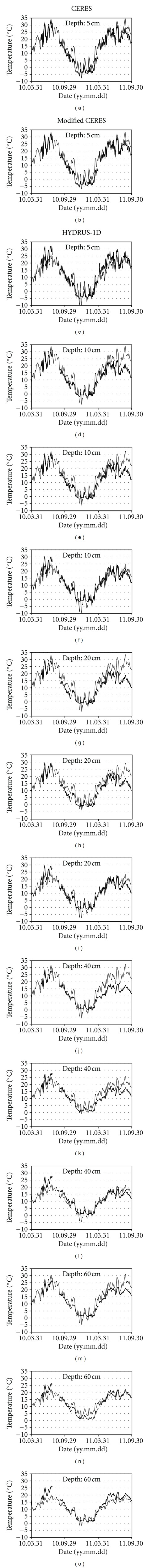
Series of measured and calculated soil temperature values at different depths, at Őrbottyán, Hungary. Thick lines—measured, thin lines—calculated.

**Figure 4:**
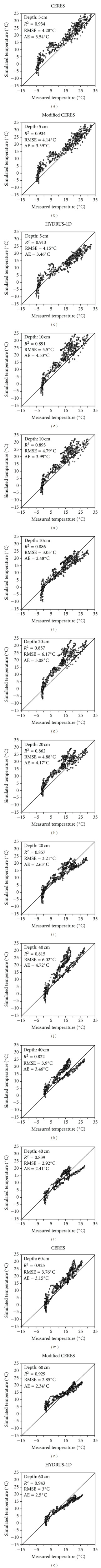
Comparison of the measured and calculated soil temperature values at different depths, at Őrbottyán, Hungary.

The calibration of (5) resulted in *c* = 4 for the newly introduced parameter. When the series of measured and calculated temperature data were analyzed, it became obvious that the original CERES considerably underestimated the soil temperature of the deeper soil layers especially in the year 2011 (Figure 3). According to the error indicators (Figure 4), the modified CERES estimated better the *T*_soil_ than the original CERES especially in the deeper layers. The calculated values of HYDRUS-1D fit the best to the measured data (Figure 4). At the top and bottom layers, the modified CERES presented similar performance indicators than those of HYDRUS-1D.

HYDRUS-1D considerably underestimated the soil temperature of the upper layers in the frosty period when the average air temperature was −7.1°C between 27/12/2010 and 04/01/2011, while the average observed and calculated *T*_soil_ was −3.4 and −6.5°C, respectively, at 5 cm depth.

 During the winter period, all models overestimated the trends of temperature changes and resulted in more pronounced oscillations of soil temperature than that of the observed values.

## 4. Discussion

The comparison of the course of measured soil temperature at two parcels (different treatments of the experiment) on a summer day highlights the effect of canopy development status on soil temperature dynamics (Figure 2). The vegetation reduces both the average soil temperature and its diurnal amplitude; therefore, considering the LAI is important in modeling.

The measured soil temperature was not below −5°C in the upper layers despite the fact that the average daily air temperature was permanently below −7°C (some days the daily minimum was below −15°C) for longer periods in December 2010 and January 2011. During this period, the site was covered with snow which reduces the effect of freezing since the depth of frost penetration is sensitive to the details of snow cover buildup [35]. As the models underestimated the actual soil temperature and overestimated the temperature oscillation within the winter period, it is obvious that all of them failed to account for the insulation effect of snow cover.

When the series of measured and calculated temperature data were analyzed, it became obvious that the original CERES considerably overestimated the summer soil temperature, while it underestimated *T*_soil_ during the winter. The minimum of the calculated soil temperature was below −5°C at 60 cm depth, while the corresponding measured temperature was +2°C. The modified CERES let less heat to be transmitted to the deeper layers resulting in lower temperatures. The calculated average error of *T*_soil_ was reduced with almost 70% compared to the original CERES at 40 and 60 cm depths.

Though HYDRUS-1D gave the most accurate soil temperature estimations, it has to be noted that this model requires more detailed inputs than CERES does. For example, CERES calculates the leaf area development, while HYDRUS-1D requires LAI data to be provided as inputs. Furthermore, HYDRUS-1D requires some thermal properties of the soil (e.g., thermal conductivity) which was estimated from the available textural information in this study. This might explain the relative moderate performance of this model.

## 5. Conclusions

Two soil temperature models using different concepts were compared in this study. The simpler empirical model was enhanced by introducing an extra parameter in one of its governing equations. The experimental results clearly showed that crop cover significantly influences the soil temperature dynamics of the upper soil layers. Therefore, considering the LAI in model calculation is indispensable. The seasonal snow cover could significantly modify the freezing of soil as it builds up an isolating layer. The simulation of the effect of snow cover should be enhanced in the investigated models. The additional parameter proposed to modify the calculation of the CERES model provided greater flexibility and resulted in better performance, though the comparison was carried out only for a very sandy soil. Further studies should be conducted to investigate the capability of the modified CERES for simulating the heat transport of more structured soils with higher clay and organic matter contents. Though the more sophisticated HYDRUS-1D provided more accurate soil temperature estimations, its superiority to CERES is not unequivocal. The considerable input requirements of HYDRUS-1D may force the users to apply parameter estimation methods which most likely decrease the model accuracy.
